# Usability, Acceptability, and Effectiveness of Web-Based Conversational Agents to Facilitate Problem Solving in Older Adults: Controlled Study

**DOI:** 10.2196/16794

**Published:** 2020-05-27

**Authors:** Matthew Russell Bennion, Gillian E Hardy, Roger K Moore, Stephen Kellett, Abigail Millings

**Affiliations:** 1 Department of Psychology The University of Sheffield Sheffield United Kingdom; 2 Department of Computer Science The University of Sheffield Sheffield United Kingdom

**Keywords:** transdiagnostic, method of levels, system usability, acceptability, effectiveness, mental health, conversational agents, older adults, chatbots, web-based

## Abstract

**Background:**

The usability and effectiveness of conversational agents (chatbots) that deliver psychological therapies is under-researched.

**Objective:**

This study aimed to compare the system usability, acceptability, and effectiveness in older adults of 2 Web-based conversational agents that differ in theoretical orientation and approach.

**Methods:**

In a randomized study, 112 older adults were allocated to 1 of the following 2 fully automated interventions: Manage Your Life Online (MYLO; ie, a chatbot that mimics a therapist using a method of levels approach) and ELIZA (a chatbot that mimics a therapist using a humanistic counseling approach). The primary outcome was problem distress and resolution, with secondary outcome measures of system usability and clinical outcome.

**Results:**

MYLO participants spent significantly longer interacting with the conversational agent. Posthoc tests indicated that MYLO participants had significantly lower problem distress at follow-up. There were no differences between MYLO and ELIZA in terms of problem resolution. MYLO was rated as significantly more helpful and likely to be used again. System usability of both the conversational agents was associated with helpfulness of the agents and the willingness of the participants to reuse. Adherence was high. A total of 12% (7/59) of the MYLO group did not carry out their conversation with the chatbot.

**Conclusions:**

Controlled studies of chatbots need to be conducted in clinical populations across different age groups. The potential integration of chatbots into psychological care in routine services is discussed.

## Introduction

### Background

The developers of psychological interventions have harnessed the internet as a delivery medium to enable increased access to evidence-based psychological therapies [1,2]. Psychological electronic therapies (e-therapies) have been defined and categorized in multiple ways that refer to properties, such as the type of technology being used or the level of therapeutic guidance involved [3]. E-therapies are typically grounded in cognitive behavioral therapy (CBT), as the protocol-driven format of CBT makes it a better fit for automation in comparison with unstructured dynamic psychotherapies [4]. There is growing evidence indicating that e-therapies are clinically equivalent to traditional face-to-face therapies in reducing the symptoms of both common mental health problems and somatic disorders [5]. This evidence is based on the outcomes achieved with working-age adults. Therefore, this leaves older adults at risk of both digital and research exclusion. For example, although older participants are rarely excluded from clinical trials of e-therapies, they account for only 3% of participants [6]. Feasibility and pilot study evidence indicate that older adults are willing to use e-therapies [7] and do find the use of e-therapies a satisfying experience [8-10]. When tested, the evidence suggests that e-therapies can be clinically effective for older adults with symptoms of depression and anxiety [11-14].

An important consideration when designing e-therapies for older adults is the user experience of the technology. User experience research typically consists of assessments of the acceptability, usability, and satisfaction of the technology being used. User experience is defined as a “person’s perceptions and responses resulting from the use and/or anticipated use of a product, system or service” [15] and usability as “the extent to which a product can be used by specified users to achieve specific goals with effectiveness, efficiency and satisfaction in a specified context of use” [15].

However, measuring the acceptability of e-therapies has typically been limited to only asking older adults to rate the acceptability of the technology before, during, and/or after using a program. Researchers have also assessed the user experience of e-therapies through measuring *treatment satisfaction*, but they have often used unvalidated questionnaires, thus bringing the results found into question [16].

Therefore, despite partially considering aspects of acceptability, usability, and satisfaction, it is rare for e-therapy studies to use the full array of international standards and associated validated instruments of usability, but there are some examples of good practice [17,18]. To maximize the reach and uptake of e-therapies for the older adults, adaptation of the methods for assessing user experience and system usability developed in engineering and computer science appears fit-for-purpose [19]. This is particularly important given the evidence that the older adults experience difficulty using e-therapies when instructions overload working memory, making it harder to effectively engage with the program [20]. Therefore, the older adults need to continually relearn how to use an e-therapy program, and on-going feelings of frustration would reduce the ratings of acceptability of the technology and risk disengagement [20].

Thus far, attempts to fully automate psychological therapies have been plagued with difficulties of low initial uptake and subsequent low adherence [21,22]. One method that has shown potential benefit for potentially increasing adherence to e-therapies is the use of conversational agents that deliver the content of e-therapies [23]. In this approach, software programs interpret and reply to lines of everyday normal language, and a therapeutic interaction is, therefore, created (ie, a conversation takes place between the client and *chatbot,* mirroring the conversation between the client and therapist). Therefore, the process of engaging with e-therapy is more personalized, dynamic, and bespoke, rather than simply following the psychoeducational exercises and self-monitoring that comprise most e-therapies.

In total, 2 conversational agents have subsequently been the focus of most research attention: ELIZA and Manage Your Life Online (MYLO), and these represent 2 differing theories and associated approaches to the treatment of emotional distress. The earliest attempt to develop a *chatbot* was by Joseph Weizenbaum in 1966. His program (*ELIZA*) was designed to mimic Rogerian counseling, a form of person-centered psychotherapy based on humanistic principles [24]. ELIZA applies simple natural language processing rules to the user’s typed inputs to respond and generate text responses in the form of subsequent questions and responses appropriately. Despite its technical simplicity and the relative transparency of its therapeutic model, ELIZA can generate convincing dialogues, and there is anecdotal evidence of therapeutic effectiveness [25]. Despite the initial interest, little progress has been made to evolve and evaluate ELIZA into a fully automatic approach for treating mental health problems [4]. Another *chatbot* called MYLO has subsequently emerged. This is an attempt to implement a fully automated technique for treating mental health problems based on the principles of method of levels (MOL) therapy [26]. MOL is a transdiagnostic form of psychological therapy grounded in perceptual control theory [27]. MYLO uses open questions to encourage users to reflect on their thoughts, feelings, and behaviors, in a way that helps users to become more psychologically flexible, and thus, more adept at reducing distress [26]. MYLO simulates an MOL-style therapeutic conversation through an automated messaging interface.

There have been 2 previous trials with student populations comparing the outcomes achieved by MYLO and ELIZA from short single-session conversations. In a pilot trial (N=48) in a student population [28], MYLO was rated as more helpful and led to greater problem resolution, but there were no differences between the conversational agents with regard to any clinical outcomes (ie, depression, anxiety, and stress). In another student study (N=213), participants were randomized in a trial to either MYLO or ELIZA before completing poststudy and 2-week follow-up measures [29]. MYLO was again rated as significantly more helpful than ELIZA, but there were again similarly no differences between the conversational agents in terms of problem resolution and clinical outcomes.

To summarize, despite developments in the reliability of system usability testing in computer science and engineering, these approaches have not been consistently adopted in the context of the development and delivery of e-therapies. In addition, where e-therapies have been developed as conversational agents, any outcome evidence has also been unfortunately limited to working-age adults’ samples. Therefore, more research is needed to investigate the clinical potential of conversational agents in the older adults.

### Objectives

This study sought to compare and contrast the system usability of 2 *chatbots* (MYLO and ELIZA) in an older adult sample and to evaluate outcomes using a randomized and controlled outcome methodology. We hypothesized that MYLO would be more acceptable, helpful, and usable than ELIZA, based on previous research [28,29], but there would be no difference in terms of clinical outcome. A secondary aim was to examine the relationship between the system usability and acceptability of the *chatbots*, particularly as Bird et al [29] specifically called for greater knowledge concerning the usability of MYLO in different groups.

## Methods

### Participants

Ethical approval was granted for the study (ref: 007599) by the University of Sheffield’s Department of Psychology Ethics Committee. A study sample was recruited from the University of the Third Age (U3A), and participation was not monetarily incentivized. The U3A is a movement that aims to educationally stimulate members who have retired from work [30]. The study was advertised over the Web via U3A websites and offline via recruitment posters placed within U3A meeting places. Inclusion criteria for the study were (1) being older than 50 years, (2) being able to read and hear clearly (with glasses or hearing aids if necessary), (3) having no medically or professionally diagnosed current mental health disorder, and (4) currently experiencing a problem causing emotional distress.

### Measures

The time points at which self-assessed measures were administered are summarized in a Standard Protocol Items: Recommendations for Interventional Trials diagram (Multimedia Appendix 1) and Table 1.

Participants provided a brief qualitative description of their *personal problems* and stated how long those problems had been occurring. Problem distress was measured on an 11-point Likert scale (from 0—not distressing at all to 10—highly distressing). Problem distress was measured at baseline, postintervention, and 2-week follow-up. Problem solvability was measured on an 11-point Likert scale (from 0—cannot be resolved to 10—easily resolved) at baseline. To measure problem resolution, participants rated on a Likert scale, at postintervention and 2-week follow-up, to what degree the problem had resolved (from 0—not resolved at all to 10—completely resolved).

**Table 1:** Summary and timeframe of measure administration.

Measure	Baseline	Postintervention	2-week follow-up
Problem distress	X^a^	X	X
Depression, anxiety, and stress scales 21	X	X	X
Problem solvability	X	—^b^	—
Problem resolution	—	X	X
Helpfulness	—	X	X
Use again	—	X	X
System usability scale	—	X	—

^a^The measure was taken at this time point.

^b^The measure was not taken at this time point.

#### Time

The time difference in minutes between the first and last timestamp of conversation logs was used to measure the duration of using the conversational agent.

#### Helpfulness

Participants rated how helpful the conversational agent was on an 11-point Likert scale (from 0—not helpful at all to 10—extremely helpful) at postintervention and at 2-week follow-up.

#### Use Again

Participants rated on an 11-point scale (from 0—most definitely not to 10—most definitely yes) the degree to which they would use the conversational agents again, but for a different problem, at postintervention and at 2-week follow-up.

#### The System Usability Scale

The system usability scale (SUS) measures perceptions of system technology and consists of a set of 10 statements scored on a 5-point scale [31]. An example item is “I found the system very cumbersome to use.” SUS has been found to have high internal consistency in a number of large datasets [32,33], and it compares favorably with other usability measures [32]. An SUS score above 68 represents an above-average usability [34]. The SUS was only administered postintervention.

#### Depression, Anxiety, and Stress Scales 21

The depression, anxiety, and stress scales 21 (DASS-21) is a 21-item scale measuring depression, anxiety, and stress over the previous week on a 4-point scale [35]. Scores can range from 0 to 21 in each domain of the scale (depression, anxiety, stress) and are calculated by summing the scores of the representative 7 items. The DASS-21 has high internal consistency (depression: 0.91, anxiety: 0.84, and stress: 0.90[35]). Participants completed the DASS-21 at baseline, postintervention, and 2-week follow-up.

### Procedure

To be involved, participants were required to either email or phone the lead researcher (MB). The researcher inputted each potential participant’s email address into a bespoke backend study management system; the system would then send participants emails containing a Web link to view the Web-based information sheet and consent form. Upon consenting, participants were sent a further email containing a set of instructions about each stage of the study, along with a Web link to allow them to begin interacting with the conversational agent (ie, participants were free to withdraw at this or any subsequent stage). Upon clicking the link, participants were taken to a set of self-assessment baseline measures within a Web-based questionnaire. After completion, the backend study management system randomly allocated, with equal probability, participants to either MYLO or ELIZA and generated the accompanying usernames, passwords, and program Web links to enable participants to access their allocated program.

The backend study management system would then email these details to the participants along with Web links to a user-guide video and usage tips Web page. The participants were given 24 hours in which they had to click the link in the email and log in to converse with their allocated conversational agent. Conversations were suggested to have a maximum duration of 20 min. After participants ended their conversation, the software presented a set of postintervention self-assessment measures within a Web-based questionnaire. Two weeks after completion, the backend study management system sent participants an email with a link to a Web-based questionnaire that contained the self-assessment follow-up measures.

### Electronic Therapy Conversational Agents

To ensure that both systems were judged on the conversation they generated and not their respective user interfaces, the visual layout and input method of ELIZA were altered to mirror that of MYLO.

#### ELIZA

The implementation of ELIZA used in this study was based on a version by cyberpsych [36], which is accessible through the Web via a website hosted by the University of Sheffield. Conversations with ELIZA mimicked Rogerian client-centered counseling and aimed to facilitate problem solving by applying the core conditions for change during Rogerian counseling [24] (ie, congruence, empathy, and unconditional positive regard). ELIZA opens the session with *Hello, let’s talk* and then adopts a consistent nondirective approach. The participants progress the conversation by typing their problems into a text input box and pressing the return key. ELIZA then responds with a question intended to maintain the conversation.

#### Manage Your Life Online

MYLO was accessed through the Web via a website hosted by the University of Sheffield. MYLO is an automated computer-based self-help program that mimics a therapeutic conversation between a client and a therapist using MOL as the change method. MYLO works by analyzing the participant’s text input for key terms/themes and responds with questions aimed at encouraging conﬂict awareness and facilitating higher levels of awareness [28]. MYLO opens the session with *Please, tell me what’s on your mind.* The participant progresses the conversation by typing their problem into a text input box and then clicking 1 of the response rating buttons. MYLO was developed by Warren Mansell at the University of Manchester.

### Statistical Analysis

The study uses sample size calculations from Bird et al’s study [29], which was a continuation of the work carried out by Gaffney et al [28]. A Cohen *d* of 0.79 was found for the baseline and postintervention comparison of distress scores of those in the MYLO group; a power analysis indicated that the minimum group size required was 19 with adequate power (0.8). Bird et al [29] found little differentiation in improvement in distress between groups (*d*=0.31). On the basis of this, the 2 conditions would, therefore, require a minimum sample size of 104. The study aimed to achieve the minimal power requirement, and a target to recruit 120 participants was set, which would result in 60 participants per group.

Data were analyzed using IBM SPSS for Microsoft Windows (version 24). The primary measure for the study was problem-related distress. DASS-21, problem resolution, time, use again, helpfulness, and system usability were secondary outcome measures.

The study used a mixed 2 × 3 analysis of variance (ANOVA), with the group (ELIZA or MYLO) as a between-participant factor and time (baseline, postintervention, and 2-week follow-up) as a within-participant variable for the primary outcome variable problem-related distress and secondary outcome measure DASS-21. Posthoc 2-tailed *t* tests were run to explore group differences using Bonferroni CI adjustment. Secondary outcome measures problem resolution, helpfulness, and use again were compared at postintervention and 2-week follow-up using ANOVA. Secondary outcome measures time and system usability were compared at postintervention using independent *t* tests that applied Bonferroni CI adjustment. To investigate the extent to which system usability was a predictor of problem resolution, helpfulness, and use again, a series of Pearson correlation coefficients were computed to assess the relationships between postintervention system usability, problem resolution, helpfulness, and use again. Simple linear regression was then carried out to determine the effect of postintervention system usability on postintervention helpfulness, use again, and problem resolution scores.

## Results

### Sample Characteristics

**Figure 1:**
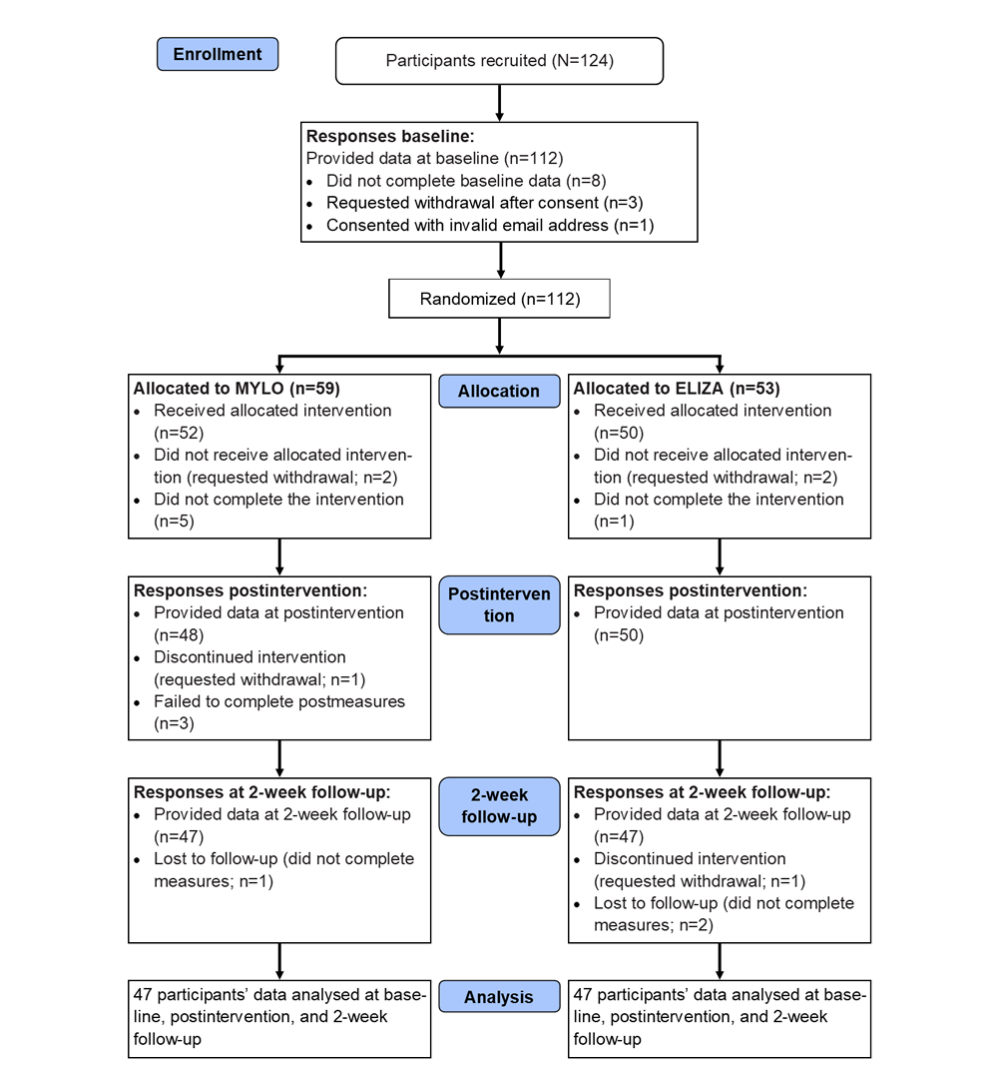
Participant flow diagram. MYLO: Manage Your Life Online.

Age of the participants ranged from 51 to 90 years, with a mean of 69.21 (SD 6.76) years, and the study sample comprised 73.2% (82/112) females and 26.8% (30/112) males. A participant flow diagram is provided in Figure 1. In total, 112 participants completed baseline measures, were randomized, and then used the conversational agents, with 98 participants providing postconversation outcomes. Of the 59 participants allocated to MYLO, 52 completed the session with a dropout rate of 12% (7/59). Of the 53 participants allocated to ELIZA, 50 completed the session with a dropout rate 6% (3/53). Across both chatbots, 92.2% (94/102) participants completed the intervention. Of those who completed the intervention, 94 (MYLO: n=47 and ELIZA: n=47) provided outcomes across all 3 time points (ie, baseline, postintervention, and 2-week follow-up). Those who completed the intervention had an average age of 68.4 (SD 6.49) years; 73% (69/94) of them were female and 27% (25/94) were male.

### Time Spent Using the Conversational Agents

The average amount of time spent engaged in conversation with MYLO was mean 24.17 min (SD 16.46), and the time spent in conversation engaged with ELIZA was mean 15.17 min (SD 8.77). On average, MYLO was used for 9 min longer than ELIZA (t_92_=3.309; *P*<.001).

### Problem Distress and Resolution

The problem-related distress and problem resolution scores for MYLO and ELIZA are reported in Table 2. There was no difference in reductions in problem-related distress over time between the 2 conversational agents (*F*_1,92_=2.39; *P*=.13). There was a significant main effect of time on distress regardless of the conversational agent (*F*_2,84_=55.85; *P*<.001). Problem distress significantly reduced between baseline and follow-up (*P*<.001), but there was no significant postintervention to follow-up reduction (*P*=.52). There was a significant interaction effect of the type of conversational agent and time on problem distress (*F*_2,84_=3.21; *P*=.04), although this was a weak effect (eta-squared=0.03). This interaction was further investigated using *t* tests. The analysis showed that there was a significant difference between interventions at follow-up (t_92_=−2.013; *P*=.05), but no significant difference was found at baseline (t_92_=0.428; *P*=.67) or postintervention (t_92_=−1.593; *P*=.12). There were also no significant differences between the 2 conversational agents regarding their abilities to enable problem resolution (*F*_1,92_=2.32; *P*=.13). There was a significant effect of time on problem resolution (*F*_1,92_=15.87; *P*<.001).

**Table 2:** Mean (SD) for measures at baseline, postintervention, and 2-week follow-up.

Outcome measures		Manage Your Life Online (n=47), mean (SD)	ELIZA (n=47), mean (SD)
**Problem distress**			
	Baseline	6.17 (1.55)	6.02 (1.81)
	Postintervention	3.68 (2.14)	4.45 (2.51)
	2-week follow-up	3.21 (2.23)	4.23 (2.67)
**Problem solvability**			
	Baseline	4.09 (2.35)	3.55 (2.25)
**Problem resolution**			
	Postintervention	2.17 (2.62)	1.51 (2.74)
	2-week follow-up	3.77 (3.29)	3.04 (2.95)
**Depression, anxiety, and stress scales 21 total**			
	Baseline	27.06 (16.18)	28.51 (19.17)
	Postintervention	20.00 (14.59)	20.64 (15.04)
	2-week follow-up	16.13 (13.91)	17.19 (14.71)
**Helpfulness**			
	Postintervention	2.94 (2.89)	1.43 (1.86)
	2-week follow-up	3.23 (2.81)	1.91 (2.21)
**Use again**			
	Postintervention	4.21 (3.14)	2.45 (2.79)
	2-week follow-up	4.43 (3.48)	2.70 (3.04)
**System usability scale score**			
	Postintervention	63.56 (17.90)	56.97 (19.46)

### Helpfulness, Use Again, and System Usability

There was a significant difference in helpfulness ratings over time between MYLO and ELIZA (*F*_1,92_=8.801; *P*=.004). At postintervention, MYLO (mean 2.94, SD 2.89) was rated as significantly more helpful (t_78.661_=3.016; *P*=.003) than ELIZA (mean 1.43, SD 1.86). There was a significant main effect of time on system helpfulness ratings (*F*_1,92_=4.627; *P*=.03). In terms of use again ratings, there was a significant difference between the conversational agents (*F*_1,92_=8.772; *P*=.004), with MYLO users postintervention more likely to use the conversational agent again for a future problem (t_92_=2.882; *P*=.005). There was no main effect of time regarding the use again ratings (*F*_1,92_=.816; *P*=.37). There were no significant differences in the postintervention system usability ratings between MYLO and ELIZA (t_92_=1.710; *P*=.09). It is worth noting that the system usability scores for both MYLO (mean 63.56, SD 17.90) and ELIZA (mean 56.97, SD 19.46) were below the cut-off for an acceptable program (ie, <68).

### Clinical Outcome

There was no statistically significant difference in DASS-21 scores over time between the conversational agents (*F*_1,92_=0.139; *P*=.71). There was a significant main effect of time on total DASS-21 scores (*F*_1.830,168.368_=33.538; *P*<.001). Total DASS-21 scores reduced significantly between baseline and postconversation (*P*<.001), between postconversation and follow-up (*P*=.02), and between baseline and follow-up (*P*<.001).

### Usability and Acceptability of the Two Conversation Agents

There were statistically significant, moderate positive correlations between MYLO system usability ratings and postintervention ratings of helpfulness (*r*_45_=0.546, *P*<.001) and interest in reusing MYLO (*r*_45_=0.542, *P*<.001), and there was a statistically significant weak positive correlation between MYLO system usability ratings and problem resolution (*r*_45_=0.420; *P*<.001; see Table 3 for details).

There was a statistically significant, weak positive correlation between the ELIZA system usability ratings and helpfulness (*r*_45_=0.344; *P*<.001) and interest in reusing ELIZA (*r*_45_=0.387; *P*<.001) see Table 4 for details). Table 4 contains the helpfulness, use again, and SUS scores for MYLO and ELIZA.

There were statistically significant, moderate positive correlations between combined MYLO and ELIZA system usability ratings and postintervention ratings of the helpfulness of MYLO/ELIZA (*r*_92_=0.473; *P*<.001) and interest in reusing MYLO/ELIZA (*r*_92_=0.487; *P*<.001; see Table 5 for details).

**Table 3:** Pearson Correlations for postintervention Manage Your Life Online ratings of system usability, problem resolution, helpfulness, and willingness to use Manage Your Life Online again.

Variables	System usability scale score	Problem resolution	Helpfulness	Use again
System usability scale score	1	0.42^a^	0.55^a^	0.54^a^
Problem resolution	0.42^a^	1	0.78^a^	0.58^a^
Helpfulness	0.55^a^	0.78^a^	1	0.79^a^
Use again	0.54^a^	0.58^a^	0.79^a^	1

^a^Correlation is significant at the .01 level.

**Table 4:** Pearson Correlations for postintervention ELIZA ratings of system usability, problem resolution, helpfulness, and willingness to use ELIZA again.

Variables	System usability scale score	Problem resolution	Helpfulness	Use again
System usability scale score	1	0.11	0.34^a^	0.39^b^
Problem resolution	0.11	1	0.39^b^	0.26
Helpfulness	0.34^a^	0.39^b^	1	0.72^b^
Use again	0.39^b^	0.26	0.72^b^	1

^a^Correlation is significant at the .05 level.

^b^Correlation is significant at the .01 level.

**Table 5:** Pearson Correlations for postintervention Manage Your Life Online and ELIZA ratings of system usability, problem resolution, helpfulness, and willingness to use Manage Your Life Online/ELIZA again.

Variables	System usability scale score	Problem resolution	Helpfulness	Use again
System usability scale score	1	0.27^a^	0.47^a^	0.49^a^
Problem resolution	0.27^a^	1	0.61^a^	0.44^a^
Helpfulness	0.47^a^	0.61^a^	1	0.78^a^
Use again	0.49^a^	0.44^a^	0.78^a^	1

^a^Correlation is significant at the .01 level.

Further tests of MYLO using simple linear regression investigated the relationship between system usability score, helpfulness, use again, and problem resolution, with system usability scores as the predictor variable.

This revealed a significant relationship between the MYLO system usability score and helpfulness (*P*<.001). The slope coefficient for system usability was 0.088, so the resolution increased by 0.088 for each extra resolution point. The *R*^2^=0.299 indicated that 29.9% of the variation in helpfulness was explained by the model containing only the system usability score⸺a significant relationship between the MYLO system usability score and use again (*P*<.001). The slope coefficient for system usability was 0.095, so the resolution increased by 0.095 for each extra resolution point. The *R^2^*=0.294 indicated that 29.4% of the variation in use again was explained by the model containing only the system usability score. There was also a significant relationship between the MYLO usability score and problem resolution (*P*=.003). The slope coefficient for system usability was 0.095, so the resolution increased by 0.095 for each extra resolution point. The *R^2^*=0.176 indicated that 17.6% of the variation in problem resolution was explained by the model containing only the system usability score.

Tests of ELIZA using simple linear regression investigated the relationship between system usability score, helpfulness, use again, and problem resolution, with system usability scores as the predictor variable. This revealed a significant relationship between the ELIZA system usability score and helpfulness (*P*=.02). The slope coefficient for system usability was 0.033, so the resolution increased by 0.033 for each extra resolution point. The *R^2^*=0.118 indicated that 11.8% of the variation in helpfulness was explained by the model containing only the system usability score. There was also a significant relationship between the ELIZA system usability score and use again (*P*=.01). The slope coefficient for system usability was 0.055, so the resolution increased by 0.055 for each extra resolution point. The *R^2^*=0.150 indicated that 15.0% of the variation in use again was explained by the model containing only the system usability score.

Finally, tests of MYLO and ELIZA results using simple linear regression investigated the relationship between system usability score, helpfulness, use again, and problem resolution, with system usability scores as the predictor variable. This revealed a significant relationship between system usability score and helpfulness (*P*<.001). The slope coefficient for system usability was 0.063, so the resolution increased by 0.063 for each extra resolution point. The *R^2^*=0.224 indicated that 22.4% of the variation in helpfulness was explained by the model containing only the system usability score. A simple linear regression was used again to investigate the relationship between system usability score and use again, with system usability scores as the predictor variable. This revealed a significant relationship between system usability score and use again (*P*<.001). The slope coefficient for system usability was 0.080, so the resolution increased by 0.080 for each extra resolution point. The *R^2^*=0.238 indicated that 23.8% of the variation in use again was explained by the model containing only the system usability score. There was also a significant relationship between usability score and problem resolution (*P*=.01). The slope coefficient for system usability was 0.038, so the resolution increased by 0.038 for each extra resolution point. The *R^2^*=0.072 indicated that 7.2% of the variation in problem resolution was explained by the model containing only the system usability score.

## Discussion

### Principal Findings

The primary aim of this study was to compare the system usability, helpfulness, and effectiveness of 2 conversational agents (MYLO and ELIZA) with regard to problem solving within a nonclinical older adult sample. This study was, therefore, a replication and extension of previous studies [28,29], but this is the first study to compare these 2 conversational agents in an older adult sample. A secondary aim was to examine the relationship between system usability and acceptability of 2 differing *chatbots*. This is an important research because the ever-increasing demand for rapid access to psychological interventions in public services means that alternative delivery methods need to be considered and tested. Such methods can replace or supplement the traditional *high intensity-low throughput* approach of traditional one-to-one and face-to-face psychological therapy delivery. The conversational agents were grounded in differing theories and approaches to the resolution of psychological distress: MOL for MYLO [26] and humanistic counseling for ELIZA [24]. However, the conversational agents tended to enable problem resolution and reductions in problem-related distress, with MYLO showing significantly lower levels of problem-related distress at follow-up. In terms of clinical outcomes, each chatbot enabled immediate reductions in DASS-21, with reductions being improved over the follow-up period.

Participants spent significantly more time using MYLO, but it is worth noting that the time spent using the program was brief in either arm (ie, an average of 20 min and this was a prompt in the instructions for using the program). Average time spent using MYLO and ELIZA is just 10-min in working-age participants [29]. These results may indicate that adults aged above 50 years are more willing to try and converse with a program of this nature. The longer MYLO conversations may be a consequence of the program’s more tailored and inquisitive questioning algorithm. In contrast, ELIZA has benefited from only limited improvements to its algorithm since its original implementation in 1966. The helpfulness and *use again* ratings of ELIZA and MYLO were significantly different, with MYLO being experienced as differentially more helpful and also more likely to be used again by participants. As MYLO was significantly more helpful, this may further explain why participants used MYLO for a significantly longer duration. These results mirror the evidence found in community working-age samples [28,29]. It may be the case that if time was allowed to be at the participant’s discretion, then ELIZA may have been rated just as helpful as MYLO.

The second aim of this study was to investigate if system usability affected the acceptability of MYLO and ELIZA when used by the older adults. Generally, correlations between MYLO system usability and problem resolution, helpfulness, and interest in reusing the system were higher than those for ELIZA. These findings indicate that *chatbot* system usability has an impact on how users perceive and rate their experience of using a conversational agent. As Web-based delivery systems do not have the benefit of a therapist to explain the rationale for certain interventions, it is essential that system usability ratings are systematically collected over the developmental iterations of the systems. This is so that when a *chatbot* goes live, it is clear and easy to use. If a system is confusing or frustrating to use, then it is highly likely to be clinically ineffective; this arguably mirrors the evidence base concerning the therapeutic alliance in general psychotherapy [37].

The findings from this study appear consistent with accepted models of system usability (eg, International Organization for Standardization 2018 [38]). Although some previous studies have also used the SUS as a measure of system usability in e-therapies [39,40], it was a strength of this study to use this validated measure and is the first usage with an older adult population using a chatbot. It is worth noting that the theoretical underpinning of the 2 conversational agents (MOL versus humanistic counseling) may have influenced the perceptions of helpfulness and, therefore, the willingness to reuse the system. High rates of attrition are assumed to be a common problem with unsupported Web-based interventions, but a meta-analysis [41] has found that the percentage of completed sessions in face-to-face CBT (83.9%) did not differ from the percentage of completed sessions in internet-delivered CBT (80.8%). The overall session completion found in this study was higher 92.2% (94/102), but this was probably due to the intervention using a single-session approach.

### Limitations and Future Directions

The study is limited by the fact that it did not recruit enough participants, and therefore, results should be considered with due caution, due to being somewhat underpowered. It is possible that the positive effects over time were due to either regression to the mean or natural recovery processes, rather than the impact of the chatbots. It is worth noting that, based on the power calculation, sufficient power was achieved for baseline to postintervention comparisons. Future studies comparing *chatbots* in clinical samples would, therefore, benefit from randomly allocating to a no treatment−passive control, to compare clinical outcomes for conversational agents against any natural recovery rate. Participants were recruited from an organization whereby membership would imply that they were open-minded to new experiences and willing to learn, and therefore, the results may not generalize to other older adults in terms of willingness to interact with a *chatbot*. It would also be useful to determine the average *chatbot* session length, when the time of the session is not recommended or limited or when there is a clinical problem being discussed.

The prompt concerning conversations needing to last approximately 20 min may have impeded deeper engagement, thus preventing problem resolution. In terms of future research, there are no published studies that investigate how the SUS interacts with other dimensions of e-therapy, such as treatment credibility, and further studies should examine this in more depth. Future studies should also assess clinical populations across the age ranges to evaluate if system usability and clinical outcomes differ between diagnoses. If the primary outcome is problem solving, then a conversational agent that follows the principles and stages of problem solving also needs to be developed and tested. The study would have benefited from a longer follow-up period, and future studies should enable short- and long-term follow-up. A possible innovation in future studies would be to adopt a patient preference trial methodology, whereby participants are offered the choice either MYLO or ELIZA (ie, to suit their preference) and those participants that are ambivalent about the choice of chatbot can be randomized.

Due to increasing referral pressure on mental health services, the flexibility of service delivery systems is important in reducing wait times for treatment, particularly in geographically remote regions. Approximately 5% to 15% of the older people also report chronic loneliness [42], and thus, *chatbots* appear to offer some potential in terms of offering conversational support to isolated older people. Talking with a conversational agent may also be particularly useful for psychological disorders involving high levels of shame and embarrassment. Indeed, the real utility of *chatbots* may be in supplementing traditional psychotherapies by reducing the number of sessions needed, because the conversational agent can provide between-session support and the therapist can focus on challenging change work during face-to-face treatment sessions. Similar models of augmenting face-to-face therapy with electronic alternatives have been discussed by Broglia et al [43]. The manner in which conversational agents could be usefully integrated into care pathways of routine psychological services needs to be explored.

### Conclusions

In conclusion, this study sought to contribute to the evidence base regarding the utility and effectiveness of *chatbots* for psychological problems. This was achieved by comparing and testing 2 equivalent systems in terms of their acceptability, helpfulness, and effectiveness using a nonclinical older adult sample. The results have proven to be both similar and different from previous studies in working-age adults; MYLO is more helpful, but neither conversational agent differentially enabled problem resolution. Future controlled studies are clearly needed to further evaluate the clinical and health economic utility of conversational agents, but the context needs to be more clinical, outcomes need to be evaluated over longer periods, and system usability needs careful consideration.

## Appendix

Multimedia Appendix 1 Standard Protocol Items: Recommendations for Interventional Trials (SPRINT) diagram.

## Abbreviations

ANOVA: analysis of variance
CBT: cognitive behavioral therapy
DASS-21: depression, anxiety, and stress scales 21
e-therapies: electronic therapies
MOL: method of levels
MYLO: Manage Your Life Online
SUS: system usability scale
U3A: University of the Third Age
