# Supplementary figures and images for: Usability, Acceptability, and Effectiveness of Web-Based Conversational Agents to Facilitate Problem Solving in Older Adults: Controlled Study
Source: J Med Internet Res. 2020 May 27;22(5):e16794. doi: 10.2196/16794 (PMC7287711; doi:10.2196/16794)

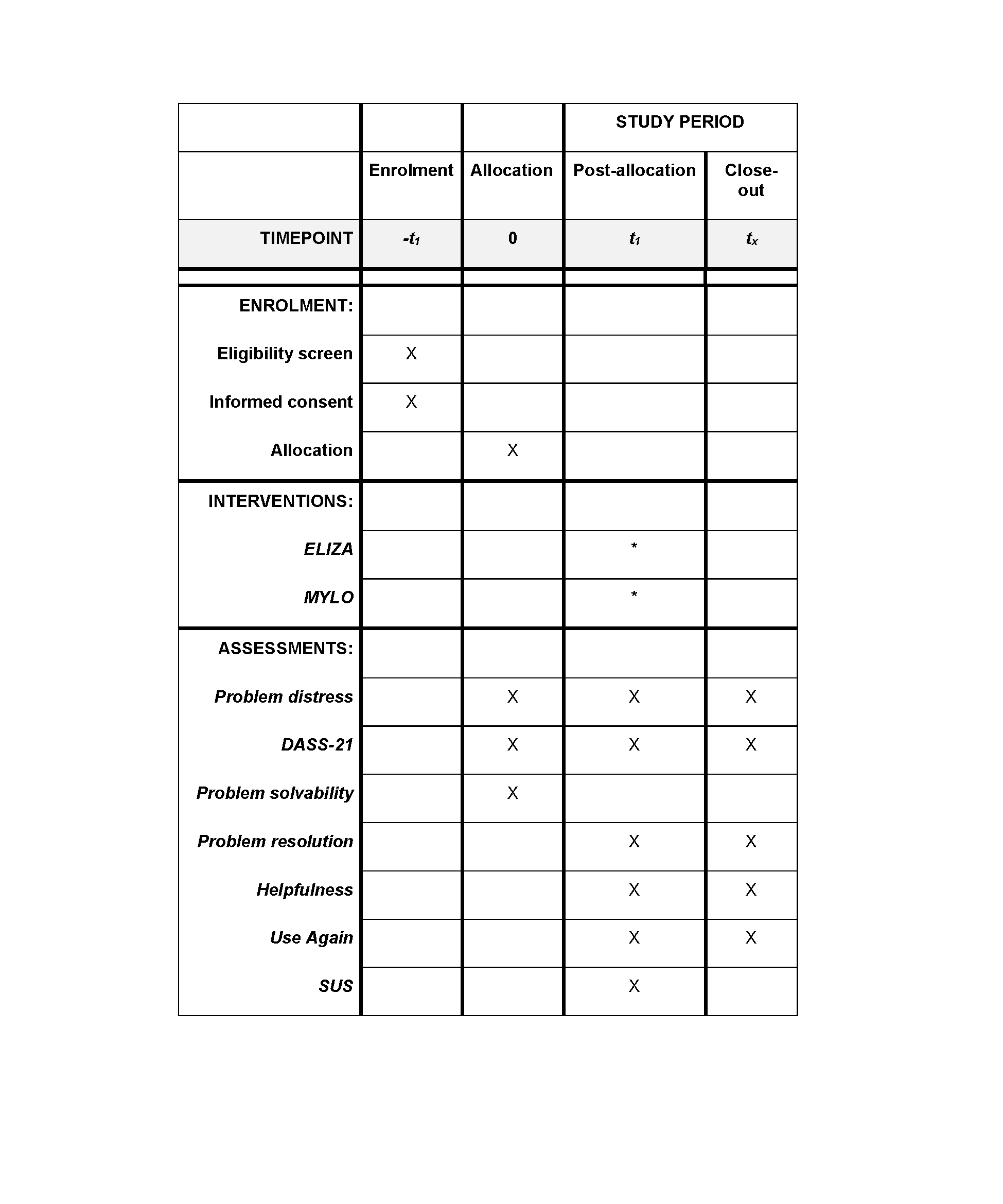

Supplement: Multimedia Appendix 1 [file jmir_v22i5e16794_app1.png]
